# Valorizations of recycled polymers/bottom ash composite for gamma attenuation and radiation shielding application

**DOI:** 10.1038/s41598-025-24552-0

**Published:** 2025-11-19

**Authors:** Khadija M. Zadeh, Maryam Al-Ejji, Deepalekshmi Ponnamma, D. A. Abdulmalik, F. Iqbal, Duha Hasnawi, Alaa Azoz, Mohammad Irshidat

**Affiliations:** 1 Center for Advanced Materials, Qatar University, P.O. Box 2713, Doha, Qatar; 2 College of Engineering, Qatar University, Doha, Qatar; 3 Department of Physics and Materials Science, College of Arts and Science, Qatar University, 2713 Doha, Qatar

**Keywords:** Recycled, Waste materials, Bottom ash, Radiation shielding, Gamma attenuation, Circular economy, Materials science, Nanoscience and technology

## Abstract

This study investigated the potential of recycled polymer (RP) /bottom ash (BA) composites for gamma radiation shielding applications and focused on the optimization of shielding performance by repurposing industrial by-products like BA from local incineration plants. Different concentrations of BA were prepared as follows: 1 wt.%, 3 wt.%, 5 wt.% and 10 wt.% were incorporated in to the RP matrix. The composites were prepared through melt blending, then compressed using a mounting press with 1 mm thickness. The mechanical properties of the prepared composites, were investigated. Thermogravimetric analysis (TGA) was used to examine the thermal stability of the prepared composites, whereas the structural morphology of the tensile samples was studied using SEM. The radiation shielding capabilities of the prepared composite sheets were examined using radioactive isotopes Cs-137 (0.662 MeV) with an activity of 5μCi. The mass attenuation coefficient for the composite sheets was measured as a function of filler percentage. The data show that the tensile strength and thermal stability of the prepared composite sheets increase up to 3 wt.% of BA reinforcement, while the elongation decreases gradually. This is attributed to the good dispersion of BA in the matrix. Higher concentrations lead to agglomeration of BA filler, resulting in decreased strength. On the other hand, the composite sample with the maximum concentration of 10 wt. % BA has good shielding characteristics, with a gamma attenuation efficiency of 74% compared with the neat polymer matrix. The obtained results recommend the use of the prepared 10 wt.% composite. The significance of this research lies in addressing the reuse of industrial waste materials by valorization waste into valuable products therefore achieving circular economy as well as a promising candidate material for gamma radiation shielding capability.

## Introduction

The growing interest in sustainable construction materials has driven research into the use of industrial wastes and by-products^1^, particularly in the development of polymer concretes and polymer composites (PCs)^2,3^ The rising use of recycled polymers, such as polyethylene, alongside industrial by-products like fly ash and bottom ash (BA), offers a pathway to reducing the reliance on expensive composite materials and lowering the overall environmental impact of the construction industry^4^. Fly ash, a by-product of coal combustion, is widely studied for its mineral composition, cementitious properties, and heavy metal content, which makes it a viable component in cementitious applications like concrete and mortar^5^. In addition to fly ash, other materials such as powder ceramics, recycled tires, carbon black, recycled sand and calcium carbonate have also been incorporated into these applications, demonstrating improvements in material properties^6–8^. One promising application is the integration of fly ash into polymers such as polypropylene (PP), polyethylene terephthalate (PET), polystyrene (PS), and high-density polyethylene (HDPE)^9–15^.

Studies have shown that ash fillers can improve tensile strength in polymer composites without negatively impacting other properties like thermal stability^16^. Additionally, the use of BA, a coarser by-product of coal combustion, in building materials is gaining attention 23, 25. Although BA lacks enhanced workability in such cases compared to fly ash, its chemical composition is similar. When ground to a finer consistency, it can serve as a pozzolan, contributing to the production of high-strength concrete^17^. However, large quantities of BA are still discarded, raising environmental concerns, particularly due to its potential to release heavy metals into groundwater, which is considered one of the main risks for water resources. Another risk that affects air quality is radiation exposure. Radiation exposure is one of the most important issues, which can lead to catastrophes as reported in the Chernobyl nuclear power plant accident. More than 30 years later, reports still provide evidence of the environmental effects of the accident that occurred in 1986^18^. Generally, ionizing radiation such as gamma and neutrons are hazardous if exposure limits exceed 20 (mSv/year), which can cause a series of health issues^19^. Therefore, it is necessary to provide shielding to reduce radiation doses resulting from neutrons and gamma exposure^20,21^, which have high energy of penetration due to short wavelength and high frequency above $30\times {10}^{20} \text{Hz}$^22^.

For gamma radiation, it interacts with matter causing three different phenomena: the photoelectric effect, where the incident energetic photon ejects a core electron called an Auger electron with emission of a characteristic x ray photon; this predominately occurs when the incident gamma photon’s energy ranges from 10 to 100 keV. The second interaction may occur is Compton scattering, where the incident photon is deflected by a shell electron with reduced gamma energy (higher wave length) causing electron ejection; this mainly occurs when the incident gamma photon’s energy ranges from 100 to 10 MeV and increases with the atomic number of the shielding material. The third process is pair production, which occurs when the incident photon’s energy is two times higher than the rest mass of the electron (0.511 MeV), producing a pair of particles: an electron and a positron^19^.

Gamma attenuation and absorbance in materials were reported in the National Institute of Standards and Technology (NIST) database for single materials such as concrete, lead, wood, and Cu^23^. Shielding is designed to combine the most effective shielding components into a single shielding composite. Several studies reported the effect of polymer/metal oxide composites as radiation shielding, as summarized in Table 1. Thabit et al. investigated the effect of adding metal oxides such as Bi_2_O_3_ reinforced glass, which improved gamma ray attenuation through the composite at different gamma ray energies^24^. Another study by Hou et al.^25^ studied the shielding properties of gamma rays through tungsten powder filled epoxy and found that higher concentrations of tungsten enhance gamma attenuation through the composite. The same observation was reported by Chang et al., who prepared tungsten/epoxy composites for gamma radiation shielding and found that the linear attenuation coefficient for gamma rays increased through the shielding composite^26^.

**Table 1:** Summary of literature on polymer composites for radiation shielding applications.

Polymer matrix	Filler	Technique	Efficiency of Attenuation (%)	Radiation Source	References
HDPE	Pozzolanic	Twin screw extruder	80%	Am-241, Cs-137, and Co-60	^45^
HDPE	Al(OH)_3_ + Pb_2_O_3_	Hot compression molding process		137Cs	^46^
UHWMDPE	MoO_3_	Hot compression	25%	Neutron shielding using 241Am/Be	^47^
HDPE PP PVA PMMA Epoxy resin	60 wt.% Bi_2_O_3_	Thermoplastic by hot press	NA	Cs-137, and Co-60	^48^
PE	B_4_C	Hot compression	60%	Neutron shielding using 241Am/Be	^49^

Up to now, no study reports the effect of recycled polyolefin reinforced by BA as a source of metal oxide for radiation shielding applications. In this study, the valorization of BA waste reinforced RP presents a sustainable solution to their environmental impact. Recycled polyethylene, being non-degradable, contributes to long-term pollution, while BA is a known source of heavy metals that can contaminate groundwater. By converting these waste streams into valuable products, the construction industry can both address environmental challenges and reduce the carbon footprint associated with Portland cement manufacturing. Efforts to replace Portland cement with alternative materials, such as coal bottom ash and geopolymers, have shown promising results^27^.

Geopolymers, formed from alumino-silicate sources like fly ash, offer an eco-friendly alternative to traditional cement due to their lower greenhouse gas emissions^28^. BA, with its similar chemical composition to fly ash, holds potential for use in geopolymer production but requires additional processing to enhance its reactivity^29,30^. This research offers promising insights into the development of eco-friendly composite materials, particularly suited for Qatar’s construction and industrial sectors. With the ability to enhance radiation shielding in buildings^31,32^, these composites can protect against harmful gamma and other radiation types. Beyond construction, these materials could be employed in the production of protective clothing and equipment designed to shield individuals from radiation exposure, making them versatile solutions for safety in both structural and personal protection contexts.

Radiation shielding, in the context of the UN’s Sustainable Development Goals (SDGs), can be related to several key areas. Specifically, it contributes to SDG 3 (Good Health and Well-being) by ensuring the use of effective radiation shielding materials^33^. The integration of these waste materials into concrete and polymer composites thus represents a dual benefit of environmental sustainability and technical advancement, making it a promising area for future research and development. This paper discusses the development of eco-friendly polymer and concrete composites reinforced with industrial by-products, highlighting their potential for effective radiation shielding and environmental sustainability.

## Materials and methods

### Materials

The RP consists of a mixture of HDPE and LDPE. These thermoplastic granules were obtained from Doha Plastic Company, located in the industrial area, Qatar. Polymers were characterized in previous work^14,15^. BA was collected from the incineration plant belonging to the Domestic Solid Waste Management Centre, Qatar. The BA composition was analyzed using XRF, which corresponds to the same composition reported in previous work^34^.

### Composite fabrication

The compounding of composite materials was carried out using a twin-screw Brabender extruder. The processing temperature was set at 200 °C, with a screw speed of 30 rpm. The polymers were added to the extruder and mixed for 3 min, after which the BA was added, and the mixture was mixed for an additional 3 min. Once the polymers were well mixed with the filler, the composites were injected. The composites were prepared according to the formulation shown in Table 2. The density of the composites was calculated for a rectangular sheet with dimensions of 0.1 cm thickness, 1 cm width, and 5 cm length. The composites were then compressed using a mounting press at 2 tons and 200 °C for 3 min, followed by cooling to room temperature.

**Table 2:** Composites formulation.

Sample Code	Composition wt. (%)		Density (g/cm^3)^
	Recycled PE (RP)	BA	
RB0	100	0	0.62 ± 0.03
RB1	99	1	0.82 ± 0.05
RB3	97	3	0.91 ± 0.09
RB5	95	5	0.96 ± 0.05
RB10	90	10	1.78 ± 0.52

### Characterization techniques

Fourier transform infrared spectroscopy (FTIR; Nicolet/FTIR 670 Thermo Nicolet) was used to investigate the structural properties of the polymer matrix at different concentrations of BA. Spectra were recorded from 400 to 4000 cm^−1^. Thermogravimetric analysis (TGA) was performed to determine the effect of BA on the thermal stability of the BA/RP composite samples. A TGA 4000 PerkinElmer thermogravimetric analyzer was used to test samples in the range of 30–700 °C at a heating rate of 10 °C /min under a nitrogen atmosphere. Thermal properties of the composites were further analyzed using Differential Scanning Calorimetry (DSC, Perkin Elmer DSC-7). DSC was used to study the crystallinity percentage and melting behavior of the composites. The degree of crystallinity (χ_c_) was calculated using Eq. 1:

1 $$\chi c = \frac{{\Delta H_{f} }}{{\Delta H_{o} W}} \times 100s$$

where ∆H_f_ is the heat of fusion of the sample, ∆ H_o_ is the heat of fusion of 100% crystalline PE (293 J/g)^35^, and W is the weight fraction of the polymer in the composite. All measurements were performed under a nitrogen atmosphere at a heating rate of 10 °C /min. Three runs were conducted: heating from room temperature to 200 °C, cooling, and a second heating, which was used to calculate the composite crystallinity. Mechanical properties of the composites were evaluated using tensile tests, which measure the force required to break specimen and the extent to which the specimen elongates at the break point. Tensile properties were measured using a universal testing machine (Instron model 6657) at a crosshead speed of 10 mm/min according to ASTM D638. Tensile modulus, tensile strength, and elongation at break were determined for samples punched to the dimensions of 50 mm length × 10 mm width × 0.5 mm thickness. 

The surface morphologies of the tensile-fractured specimens were coated with a thin layer of gold to enhance conductivity and prevent charging. Fractured specimens were observed using scanning electron microscopy (SEM, NOVA Nano SEM450, FEI, Thermo Fisher, Netherlands).

Experimental measurements were also carried out to study the gamma ray attenuation properties of BA/RP composites with different BA concentrations. Samples were placed in front of a Cs-137 gamma source with a radioactivity of 5µCi, and a Geiger Muller (GM) detector was used to measure radiation in Counts per Minute (CPM). Gamma radiation interacted with the BA/RP composite samples, resulting in scattering and absorption, while the remaining photons were transmitted to the detector for counting. The mass attenuation coefficient of the composites for gamma radiation was calculated using Eq. 2:

2 $$I={{I}_{^\circ } e}^{- \frac{\mu }{\rho }}$$

where µ is the linear absorption coefficient, $\rho$ is the density of the sample as an absorber, and I_o_ is the radiation intensity without the sample.

## Results and discussion

### FTIR and thermal studies

**Fig. 1:**
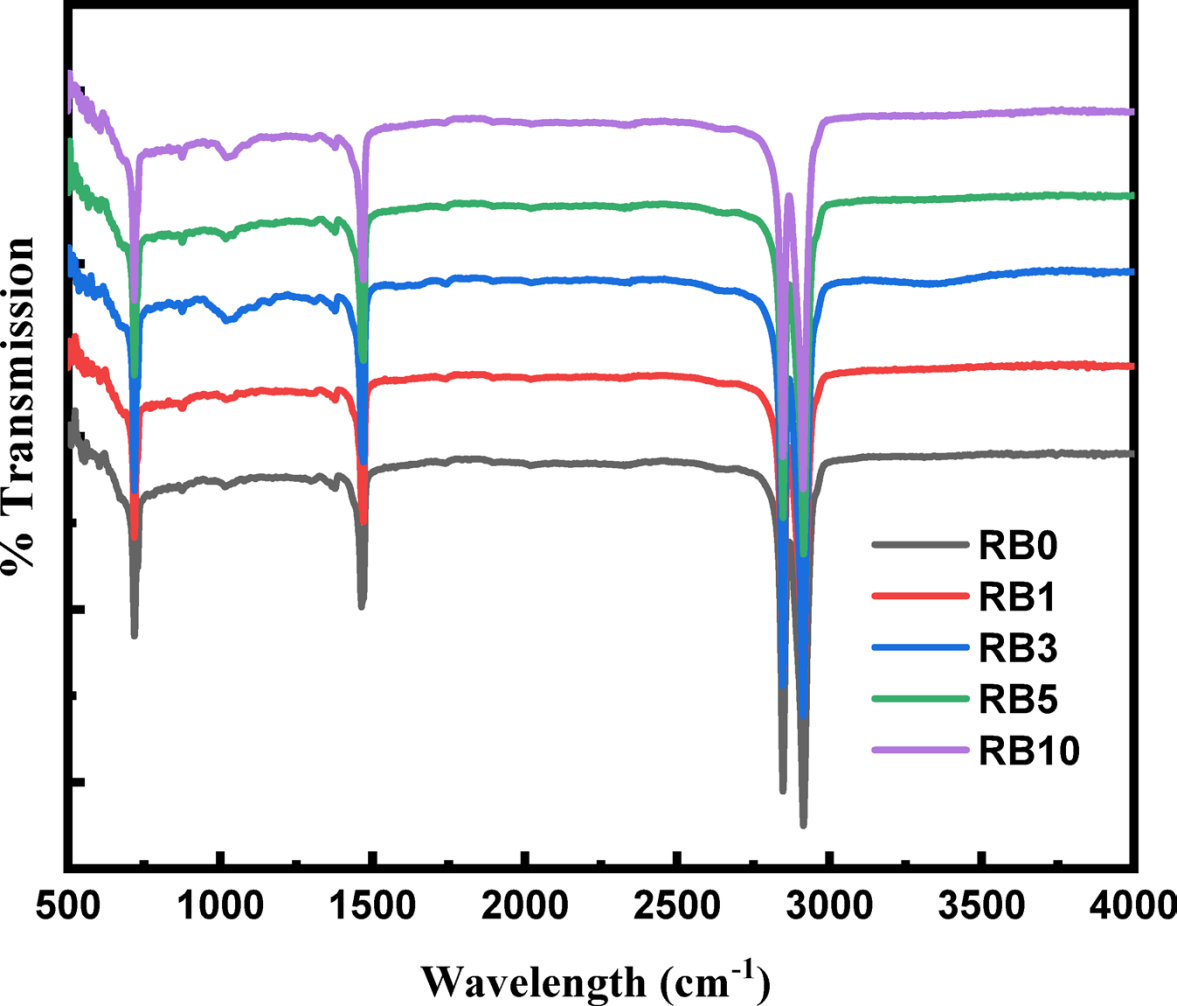
FTIR spectra of RP/BA composites.

The FTIR technique was employed to characterize the presence of functional groups of metal oxides, as shown in Fig. 1. It illustrates the transmittance peaks of recycled polyethylene (RB0), which are located at 2911 cm^−1^, 2874 cm^−1^, 1500 cm^−1^, and 718 cm^−1^. New peaks were observed in recycled polymer samples with different loadings of BA, illustrated as follows: a band near 800 cm^−1^ ascribed to the presence of the O–Si–O bending mode^36^, followed by a stretching band appearing near 1000 cm^−1^, attributed to the vibration of the CaO group, which was confirmed by XRF as the majority component of BA^37^. Although the BA concentration was increased, a clear stretching band was observed only at RB3, which may be attributed to good interaction and dispersion of CaO as a filler within the polymer matrix. In other words, it acts as an efficient nucleating agent^38^.

**Fig. 2:**
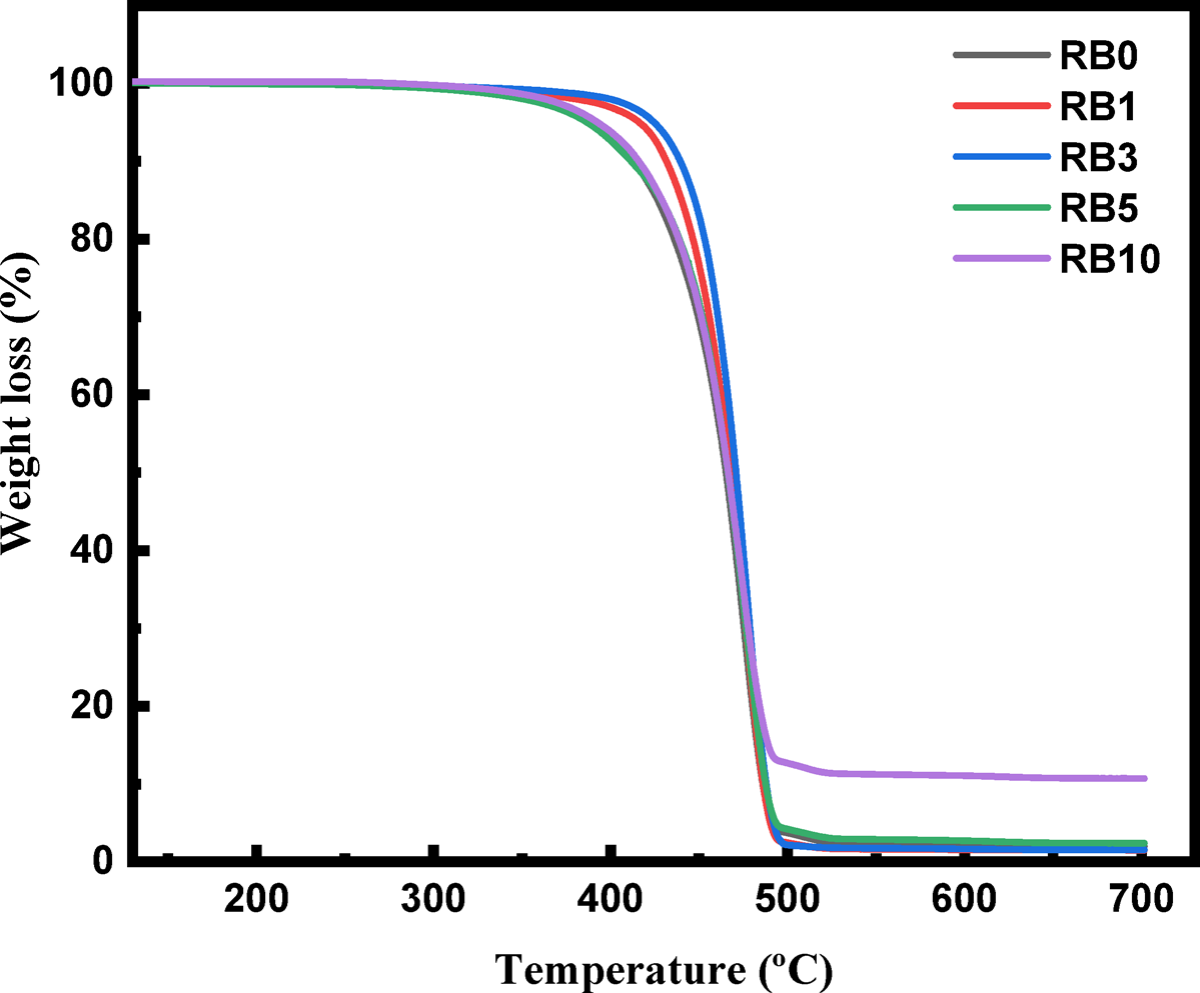
TGA thermogram of recycled polymer and RP/BA composites.

Thermal stability of the polymer composites was evaluated using TGA, as shown in Fig. 2. The curves for the BA/RP composites all show a single step of degradation with remaining residue at 700 °C. The addition of BA caused an increase in thermal stability at T_10_, observed for RB1 and RB3, indicating good dispersion between filler and matrix, which led to enhanced thermal stability^39^. This enhancement is attributed to the heat capacity of the filler, which spreads the heat energy and prevents its localization, thereby delaying the degradation of the polymer matrix.

**Fig. 3:**
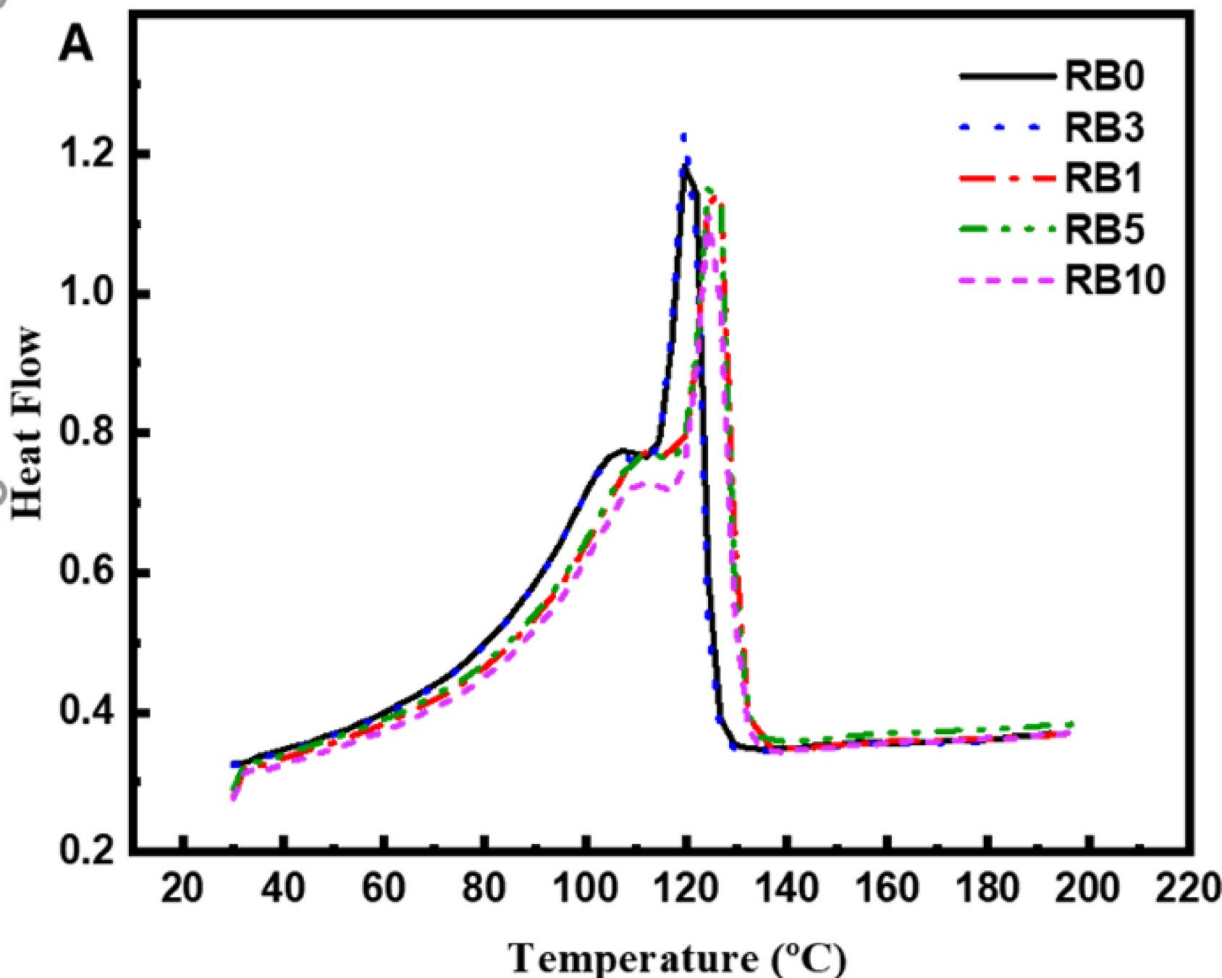
DSC heating flow of recycled polymers containing different percentages of BA.

The DSC results for all samples are shown in Fig. 3. Two melting peaks were observed for all samples, indicating that the recycled polymer contains a mixture of HDPE and LDPE, represented by melting temperatures of 125.5 °C and 110 °C, respectively. BA content did not affect the melting temperatures of the composites; however, the heat enthalpy showed changes, reflecting an influence on crystallinity. Table 3 summarizes the effects of BA on crystallinity.

**Table 3:** Thermal characteristics of the composites from DSC.

	Hm(J/g)	Tm (ºC)	Hc(J/g)	Tc (°C)	Xc %
RB0	99.67	125.5	85.7	111.5	14.3
RB1	97.5	125.1	84.7	111.7	22.3
RB3	103.3	125.2	85.7	111.3	26.2
RB5	94.9	124.5	84.3	111.6	15.8
RB10	95.43	125.7	82.3	111.6	15.1

### Tensile testing and morphology examination

The tensile properties of the composites are presented in Table 4. It is clear from the results that the addition of BA improves the tensile strength and modulus of the composites compared with the neat polymer (BR0). The optimum increase in concentration was observed for BR3, where BA acted as a nucleating agent^39^. Consequently, the strength and modulus increased, while the elongation at break decreased gradually due to the materials becoming stiffer compared with the ductile neat polymer. A similar observation was reported by Kastiawan et al.,^40^. At higher BA contents, a gradual decrease in tensile strength was observed, which is attributed to the agglomeration of BA. Therefore, the optimum concentration for mechanical strength was obtained at 3 wt. % BA in the polymer matrix.

**Fig. 4:**
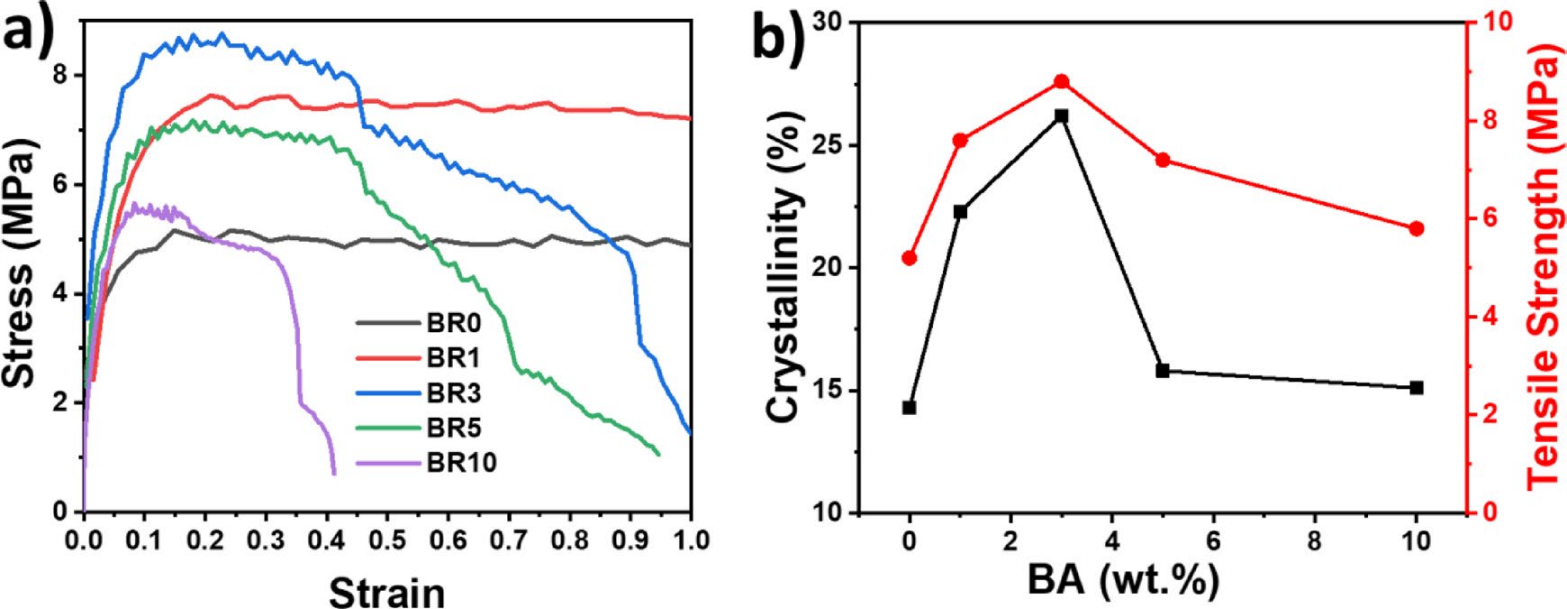
(**a**) Stress–Strain curves and (**b**) comparison of crystallinity and tensile strength for composites with different concentrations of BA.

Figure 4a presents the stress–strain curves of the composites, showing the improvement in tensile strength and modulus with BA addition up to the optimum concentration, beyond which the strength decreases due to agglomeration. Figure 4b illustrates the correlation between crystallinity and tensile strength, where both parameters follow a similar trend with increasing BA content, confirming that controlled filler dispersion enhances both mechanical and structural properties of the composites. Islam et al. reported the same observation for metal oxide-reinforced high-density polyethylene, confirming that high loading of metal oxide nanoparticles may cause agglomeration, which leads to a decrease in mechanical strength^41^.

**Table 4:** Tensile properties of BA composites.

	Tensile strength (MPa)	Tensile modulus (MPa)	Elongation at break (%)
BR0	5.2 ± 0.2	57.4	306
BR1	7.6 ± 0.3	76.3	210
BR3	8.8 ± 0.2	141.9	114
BR5	7.2 ± 0.1	99.8	96
BR10	5.8 ± 0.2	137.7	42

**Fig. 5:**
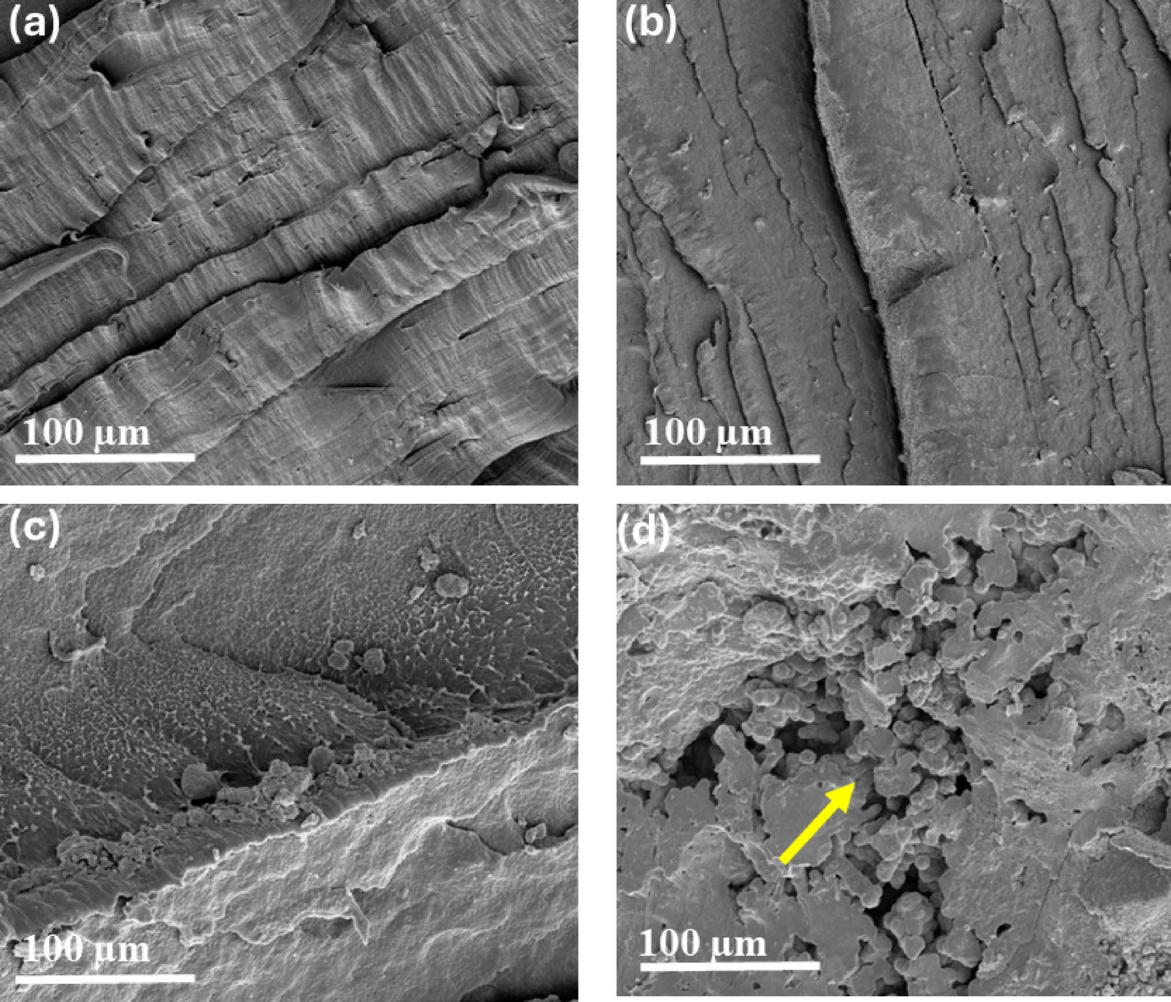
Tensile-fractured surfaces of the samples: (**a**) RB0 and (**b**) RB5 at 1000 × magnification, and (**c**) RB3 and (**d**) RB10 at 5000 × magnification.

The SEM images of the tensile-fractured surfaces of the neat polymer and composites with different BA concentrations are shown in Fig. 5a–d. Figure 5a shows the fractured surface of neat polymer, in which ductility is indicated by the elongated matrix in the form of fibrils. In Fig. 5b, the addition of 5 wt.% BA in the polymer matrix produced a rigid morphology with fewer elongated fibrils, indicating increased rigidity compared with the neat polymer. Figure 5c shows well dispersed BA in the polymer matrix, resulting in improved tensile strength. However, Fig. 5d reveals agglomeration of BA in the polymer matrix, which led to non-coalescence of BA particles with the polymer, causing voids and consequently reducing the mechanical properties^15^. This microstructural observation also explains the decrease in thermal stability at higher BA loadings, as agglomerated particles accelerate polymer decomposition, highlighting the consistent relationship between the SEM morphology, mechanical performance, and thermal behavior of the composites.

### Gamma ray attenuation

**Fig. 6:**
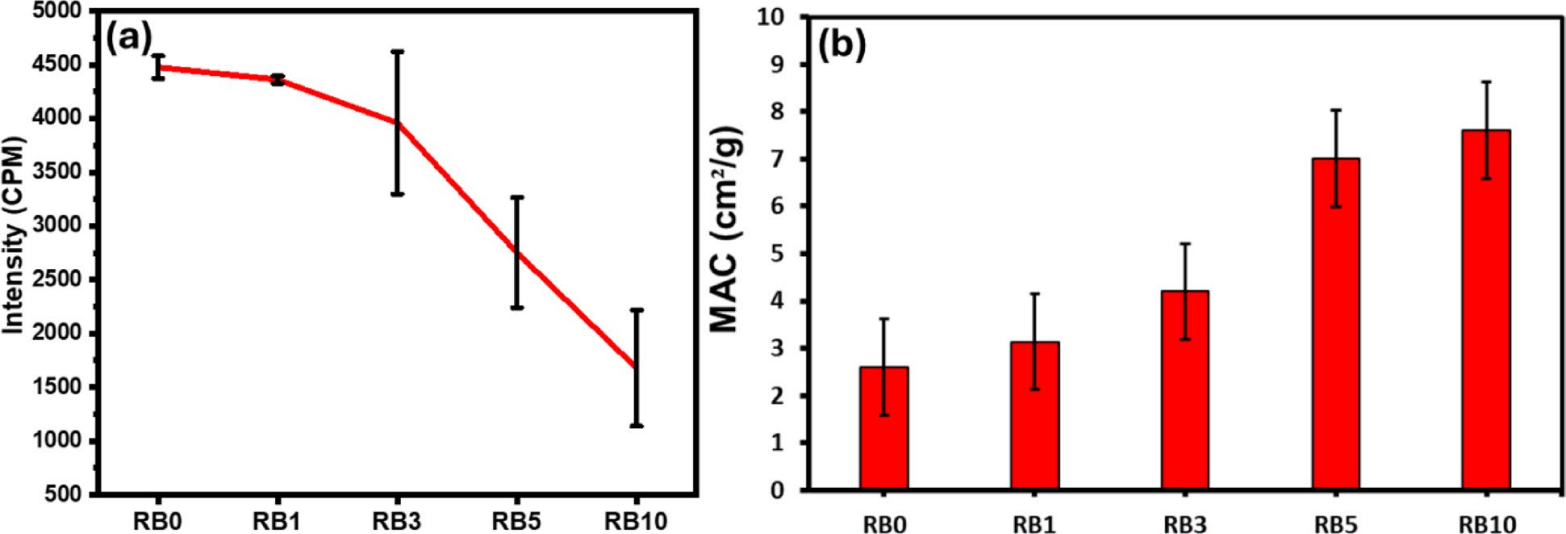
(**a**) Radiation intensity of neat polymer (RB0) compared with BA-filled composites; (**b**) Mass attenuation coefficient of the same composites.

The intensity of the gamma source detected by the GM detector (I_0_) was 6500 CPM. For all samples with 1 mm thickness, the radiation intensity was recorded as shown in Fig. 6a. The neat polymer (RB0) shielding reduced the intensity to 4500 CPM, and a gradual decrease in radiation intensity was observed with increasing BA content, reaching 1650 CPM for RB10. RB5 was identified as the half-value layer (HVL), defined as the thickness of material required to reduce the initial radiation intensity by half. This indicates that 5 wt.% of bottom ash is sufficient to reduce the radiation flux by 1 mm thickness. These results demonstrate a significant improvement in the performance of the composites as radiation shielding materials.

The improvement arises from the presence of metal oxides in BA, such as SiO_2_, CaO, and PbO_2_, which interact with gamma rays and disrupt their penetration through the composite. The attenuation depends on several factors, including photon energy, sample thickness, the atomic number of the matter, and mass attenuation coefficient of the shielding material.

The mass attenuation coefficient of the polymer composites at different BA concentrations was theoretically calculated using Eq. (3):

3 $${\mu }_{m}=\frac{\mu }{\rho }=\frac{1}{\rho x} \text{ln}(\frac{{I}_{o}}{I})$$

where µ is the linear attenuation coefficient,$\rho$ is the density of the composite, µ_m_ is the mass attenuation coefficient, and $x$ is the thickness of the composite.

Figure 6b illustrates the variation of the mass attenuation coefficient of the composites, which ranged from 2.6 cm^2^ /g for the neat polymer (RB0) to 7.6 cm^2^/g for RB10. This confirms that higher concentrations of BA with metal oxides provide stronger resistance to photon penetration, thereby reducing the number of transmitted gamma photons detected by the GM detector.

The composite shielding efficiency for γ-radiation was calculated using Eq. (4):

4 $$\text{Radiation shielding efficiency }\left(\text{\%}\right)=\frac{{\text{I}}_{\text{o}}-\text{I}}{{\text{I}}_{\text{o}}}\times 100\text{\%}$$

where ${I}_{o}$ is the radiation intensity without shielding, and $I$ is the radiation intensity with the composite shield. The shielding efficiency increased up to 75% for RB10 compared with the neat polymer (RB0).

Similar findings were reported by Naeema et al., who evaluated the mass attenuation coefficients of metal oxide nanoparticle-doped polypropylene composites against gamma and neutron exposure, showing that polypropylene with higher CdO concentrations exhibited optimum shielding performance with ~ 75% efficiency^42^. Belgin and Aycik also observed that increasing filler content in linear low-density polyethylene significantly reduced radiation intensity^43^. Additionally, Frahat et al. investigated fly ash and geopolymer paste in cementing materials and demonstrated their potential for radiation protection^44^.

The current study presented here confirms that BA can act as an effective gamma radiation barrier when incorporated in to polymer matrices. The results highlight that attenuation efficiency can be tailored over a wide range of composites by understanding the fundamental interactions of gamma rays with matter.

**Fig. 7:**
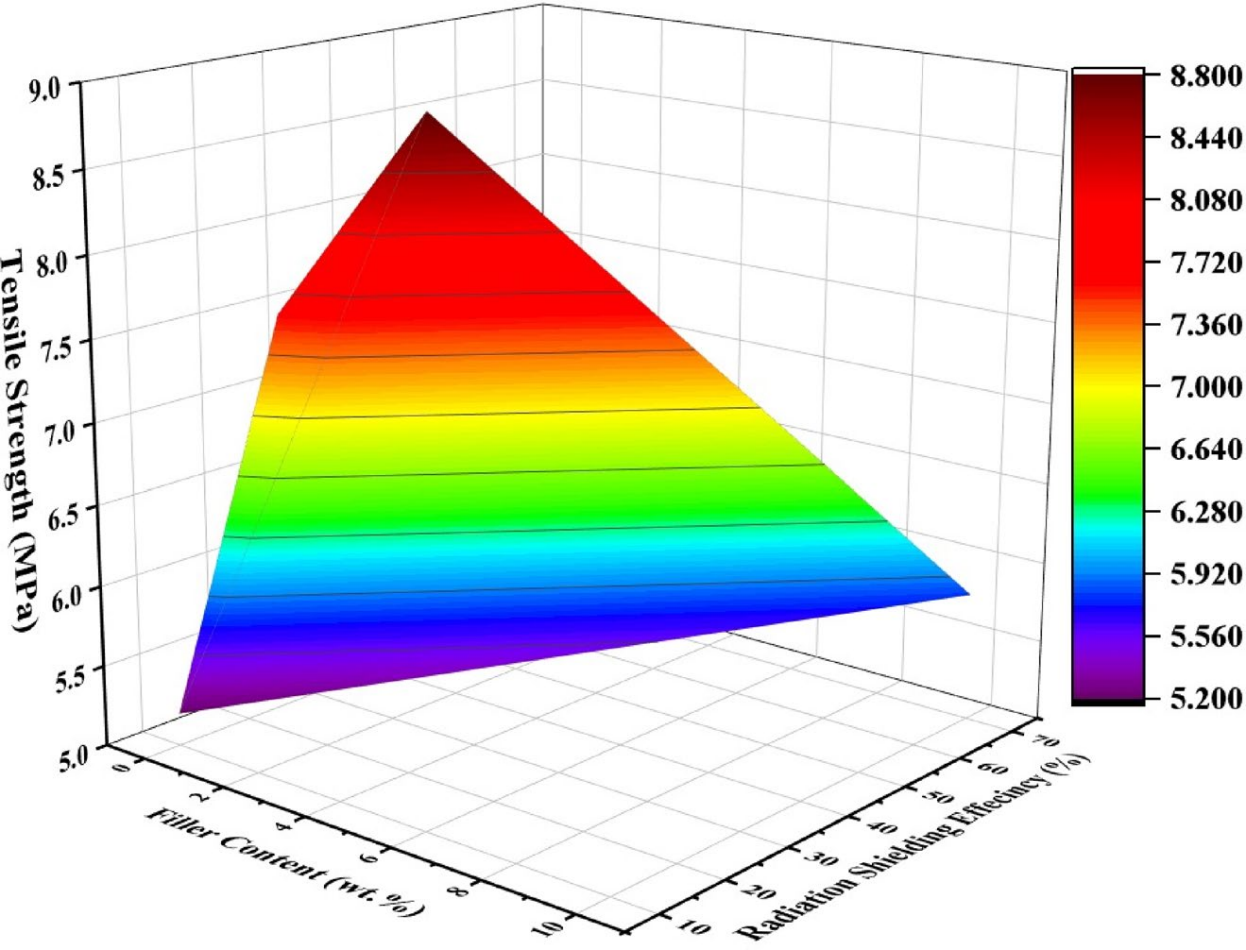
Optimization of filler content, tensile strength, and gamma shielding efficiency.

Figure 7 presents a 3D plot illustrating the relationship between BA filler content, tensile strength, and radiation shielding efficiency. The graph clearly shows that both tensile strength and shielding efficiency improve with increasing BA content up to an optimum level, beyond which the strength begins to decline due to filler agglomeration, while shielding efficiency continues to increase. This combined visualization highlights the trade-off between mechanical performance and radiation attenuation, providing an effective tool to identify the optimum BA concentration for balanced composite properties.

## Conclusions

This study examined the radiation shielding and thermal performance of recycled polymer/bottom ash composites. It was found that increasing bottom ash content enhances thermal stability, as the metal oxides act as barriers to polymer decomposition. The BA filler, incorporated into recycled polyethylene (a major waste product in Qatar), demonstrated improved radiation shielding compared to conventional lead-based materials, offering a lighter and more sustainable alternative. The composite’s mechanical and thermal properties were analysed, and gamma ray attenuation was measured using a GM detector. Composites with varying BA concentrations (0–10 wt.%) were prepared, revealing that tensile strength and tensile modulus increased with BA content, thereby enhancing stiffness. DSC results confirmed increased crystallinity, while elongation decreased, indicating greater brittleness. Scanning electron microscopy corroborated these findings. Thermal stability was optimized at 3 wt.% BA, while gamma ray absorption improved significantly at higher BA concentrations, highlighting its effectiveness in radiation shielding. This research provides valuable insights into the development of sustainable composite materials for applications in Qatar’s construction industry and beyond. These composites exhibit strong potential for radiation protection in buildings, offering effective shielding against gamma and other types of radiation. Furthermore, their versatility extends to human protection, including potential use in protective clothing and gear to shield individuals from radiation exposure in hazardous environments.
